# Supplementary figures and images for: Curcumin suppresses tumorigenesis by ferroptosis in breast cancer
Source: PLoS One. 2022 Jan 18;17(1):e0261370. doi: 10.1371/journal.pone.0261370 (PMC8765616; doi:10.1371/journal.pone.0261370)

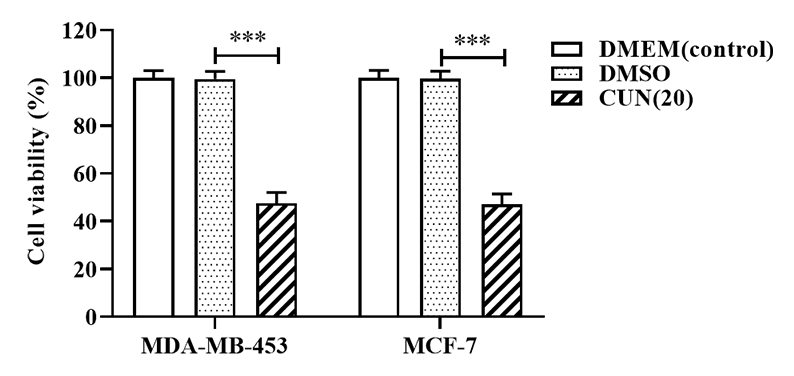

Supplement: S1 Fig — The CCK-8 assay was performed to assess cell viability in both BC cell lines treated with curcumin or DMSO. ***P<0.001, compared with the DMSO group. CUN, curcumin; CCK-8, cell counting kit-8; BC, breast cancer. (TIF) [file pone.0261370.s001.tif]

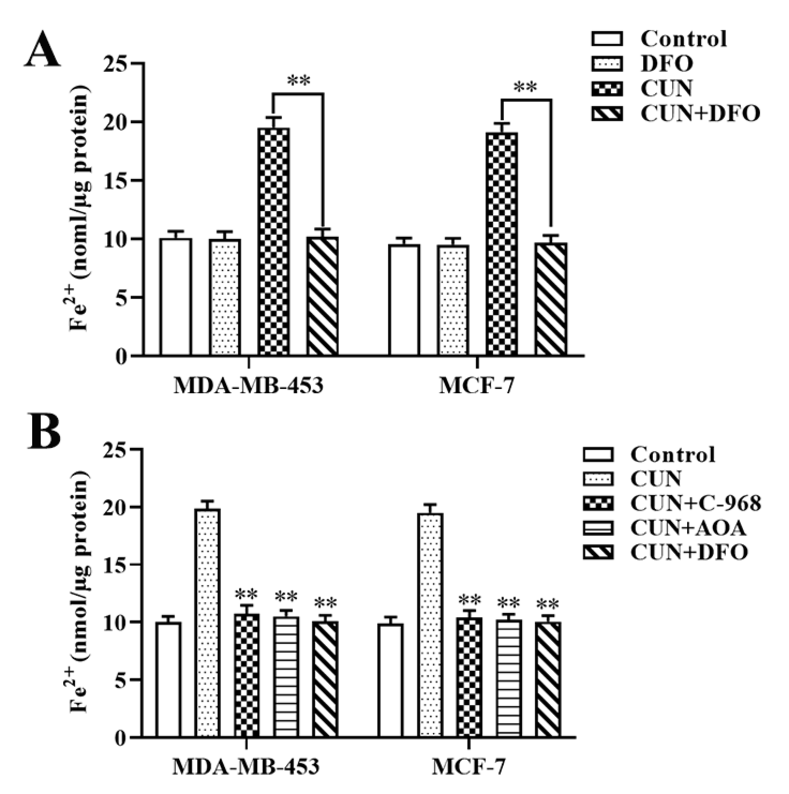

Supplement: S2 Fig — Both BC cells were preincubated with DFO (50 μM), for 2 h followed by CUN treatment for 48 h. A, B: The intracellular Fe2+ expression in both BC cell lines was measured by an iron assay kit. **P<0.001, compared with the CUN group. CUN, curcumin; BC, breast cancer; DFO, deferoxamine. (TIF) [file pone.0261370.s002.tif]

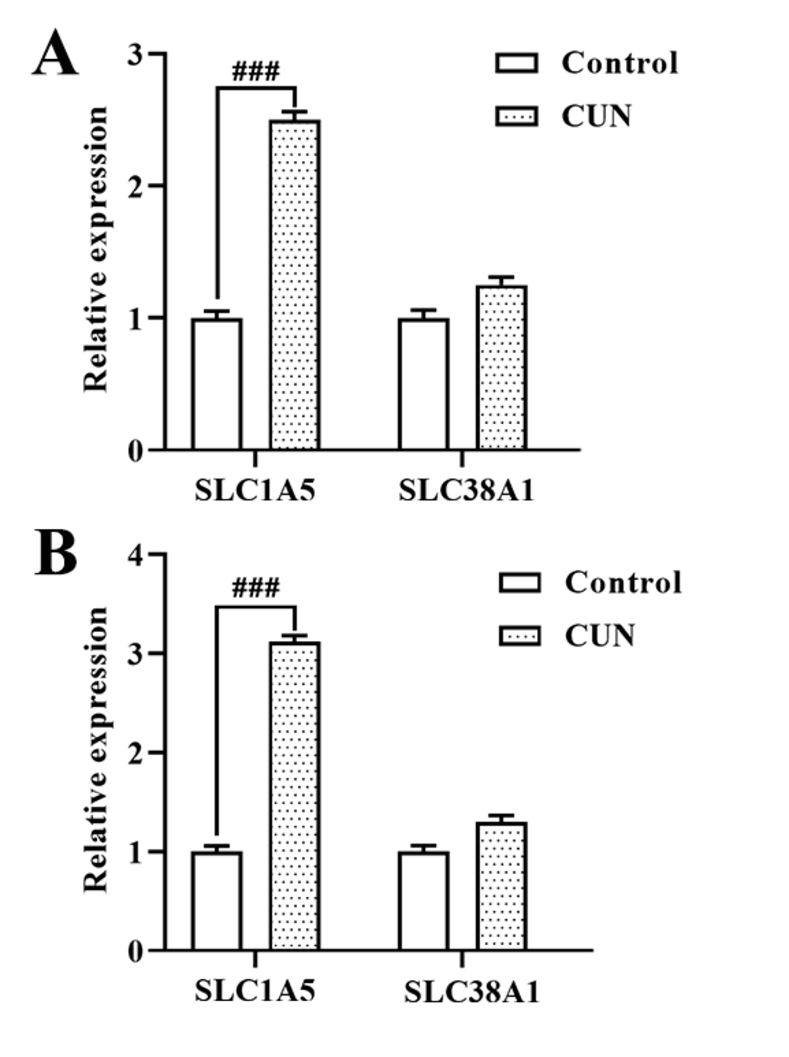

Supplement: S3 Fig — A and B: qRT-PCR was used to examine the mRNA expression levels of SLCA5 and SLC38A1. ###P<0.001. compared with the control group. (TIF) [file pone.0261370.s003.tif]
