# Supplementary material for: Curcumin suppresses tumorigenesis by ferroptosis in breast cancer
Source: PLoS One. 2022 Jan 18;17(1):e0261370. doi: 10.1371/journal.pone.0261370 (PMC8765616; doi:10.1371/journal.pone.0261370)
Supplement: S1 Raw images — (PDF) [file pone.0261370.s004.pdf]

Figure 3C

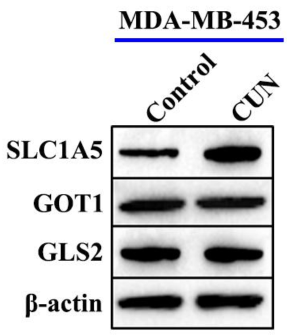

cropped

β-actin

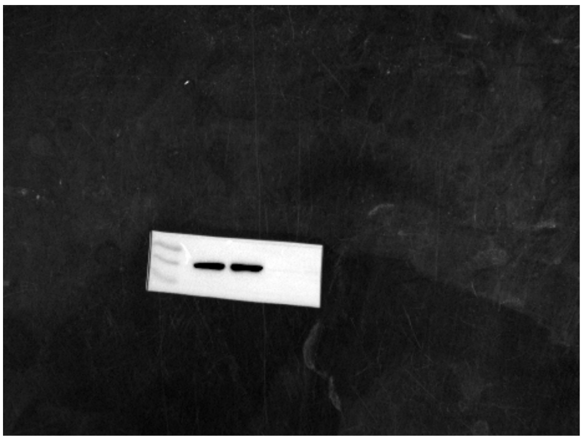

GLS2

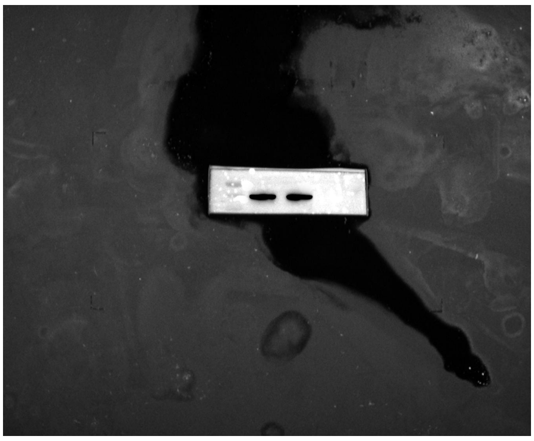

GOT1

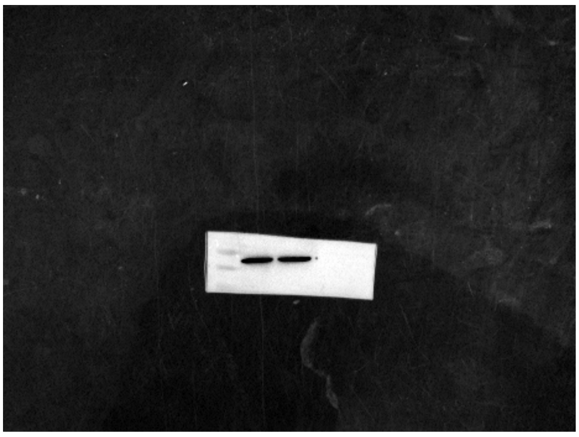

SLC1A5

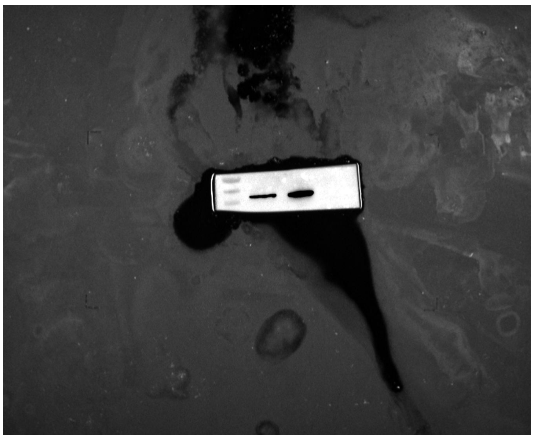

uncropped

Figure 3D

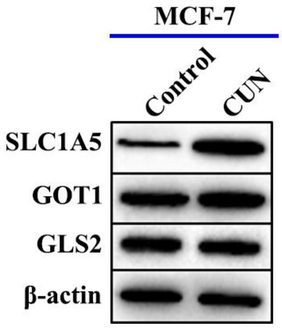

cropped

β-actin

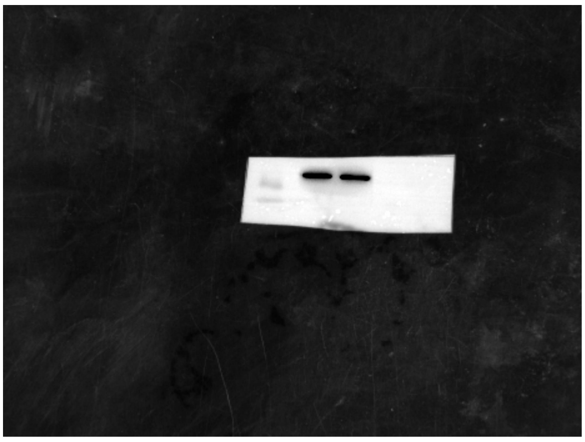

GLS2

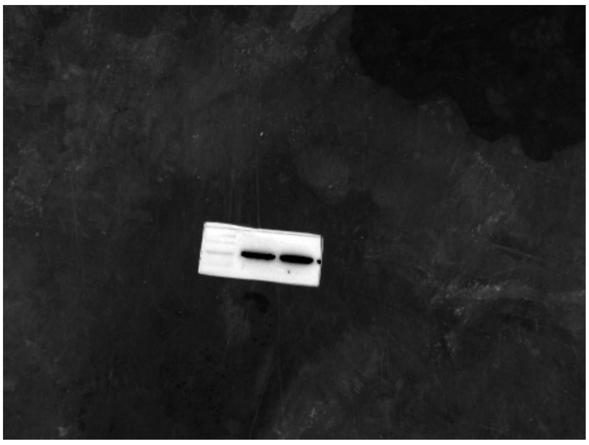

GOT1

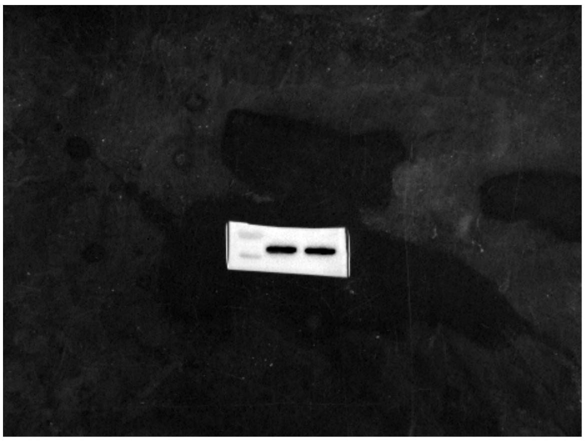

SLC1A5

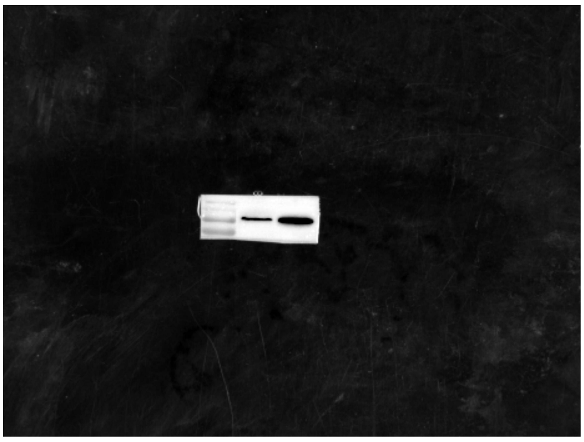

uncropped

Figure 4A

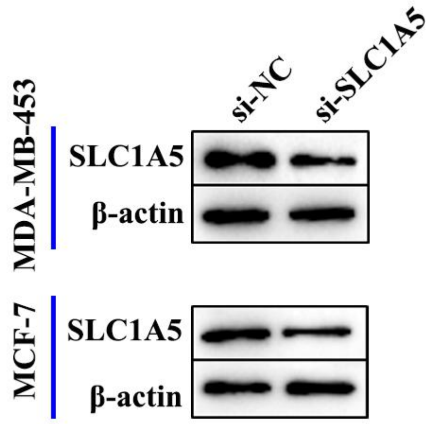

cropped

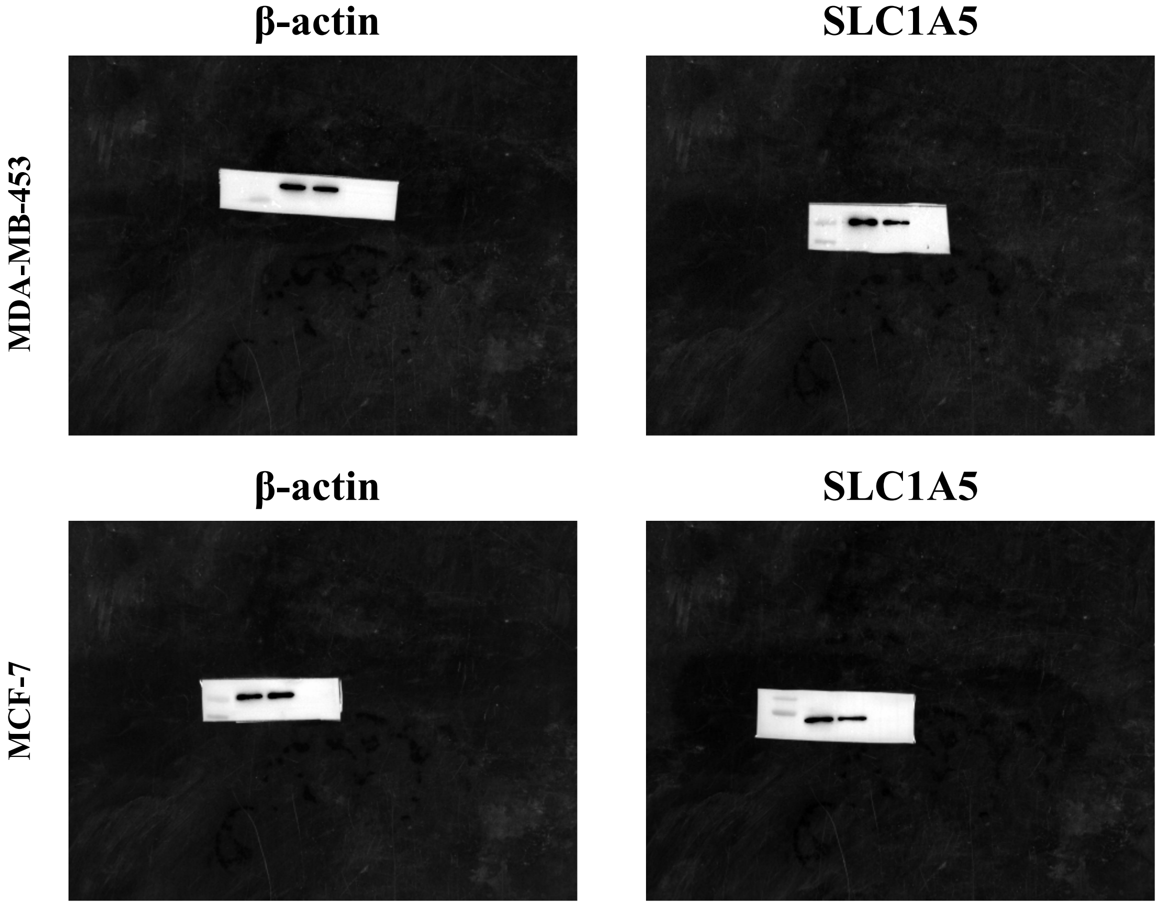

uncropped
