# Supplementary material for: Curcumin suppresses tumorigenesis by ferroptosis in breast cancer
Source: PLoS One. 2022 Jan 18;17(1):e0261370. doi: 10.1371/journal.pone.0261370 (PMC8765616; doi:10.1371/journal.pone.0261370)
Supplement: Attachment — Submitted filename: Response to Reviewers.docx [file pone.0261370.s005.docx]

**Reviewer #1:**

**Question #1:** Although the authors showed that curcumin suppressed viability of breast cancer cells and tumor growth at relatively high concentrations, it is also toxic to non-tumor cells such as mouse hippocampal HT22, PC12 and NIH-3T3 cells (Hirata et al., ACS Chem Neurosci 11, 76-85, 2020; Meng et al., PLOS ONE 9, e85570, 2014; Zhang et al., Mol Immunol 116, 191-198, 2019). Therefore, the cytotoxic effect of curcumin is not specific for breast cancer cells.

**Response #1:** Thanks for your nice comments on our article. Recently, several studies have demonstrated that curcumin had anticancer and chemoprevention effects on breast cancer through inhibiting cancer proliferation [1] and tumor metastasis [2], as well as enhancing the sensitivity of breast cancer cells to chemotherapeutic drugs [3-5]. Moreover, our data showed that 2-50 μM curcumin significantly reduced the viabilities of breast cancer cell lines (MDA-MB-453 and MCF-7). Therefore, the above results indicated the curcumin serves as an adjunct therapy in breast cancer.

**Question #2:** Line 61-62. This sentence is inaccurate, or misleading information.

**Response #2:** We rewrote this sentence as “It was reported that danshen, a traditional Chinese medicine, improved survival of patients with breast cancer and induced ferroptosis and apoptosis of breast cancer cells”. (Line 62-63, page 3 in this revision.)

**Question #3:** Lin et al. analyzed a cohort of 79,335 patients with breast cancer between 2000 and 2010 and showed that danshen improves survival of breast cancer patients. They also showed that dihydroisotanshinone I (DT), a pure compound present in danshen, inhibits the growth of breast carcinoma cells, including MCF-7 and MDA-MB-231 and induces both apoptosis and ferroptosis in those cultured cells. DT-induced ferroptosis is characterized by reducing GSH content and GPX4 protein, and increasing lipid peroxidation. Therefore, there is no direct evidence that danshen prolonged the survival of patients with breast cancer by inducing ferroptosis.

**Response #3:** In this study, the results showed that the use of Danshen ≥84 g was highly associated with decreased mortality (the adjusted HR of Danshen ≥84 g users was 0.54 [95% CI, 0.46–0.63] (p <0.001). Moreover, the use of Danshen for >28 days remained highly associated with decreased mortality (the adjusted HR of Danshen users for >28 days was 0.55 [95% CI, 0.49–0.62] (p <0.001). Thus, these data demonstrate the protective effects of a higher dose or longer use of Danshen for patients with breast cancer.

**Question #4:** Figure 2D, Figure 3H:

(1). According to these results, curcumin increased ferrous ion levels in MCF-7 and MDA-MB-453 cells. However, curcumin has effective ferrous ions chelating capacity (Ak and Gulcin, Chemico-Biol Int 174, 27-37, 2008). In fact, curcumin reduces ferrous ions in cultured cells (Hirata et al., ACS Chem Neurosci 11, 76-85, 2020) and in in vitro assay (Shome et al., Biotechnology and Applied Biochemistry 68, 603-615 Biotechnology and Applied Biochemistry, 2021) at concentrations similar to those reported in this study. The authors need to examine the effect defferoxamine on ferrous ion levels as a positive control.

**Response #4(1):** As suggested, we examined the effect deferoxamine (DFO) on the curcumin-induced increase in intracellular iron, and the results as showed in supplementary Fig. 2. We added this information in line 210-212, page 9 in this revision.

(2). Please check whether the unit of ferrous ion is correct.

**Response #4(2):** As suggested, we checked the unit of ferrous and revised this error in Figs 2 and 3 in this revision.

(3). The relative of intracellular Fe^2+^ expression …: Intracellular Fe^2+^ level in both …

**Response #4(3):** We corrected the “The relative of intracellular Fe^2+^ expression” into “The intracellular Fe^2+^ level”. (Line 483 and 493, page 17 in this revision)

**Minor points:**

**Question #5:** Figure 1: Is the concentration of erastin 10 nM?

**Response #5:** Thanks for your carefully checking. In this study, the concentration of curcumin in both breast cancer cells was 10 nM.

**Question #6:** Figure legends: The number of samples used in the experiments and statistics needs to be clarified.

**Response #6:** We have added the detailed information for the samples used in the figure legend in this revision. (Line 477, 485, 494, 504 and 513, page 17-18 in this revision)

**Reviewer #2**

In this article, the authors performed a series of in vitro and in vivo experiments to find out novel anti-tumorigenic mechanistic insight of curcumin. And they revealed that curcumin administration induced ferroptosis by enhancing the levels of lipid reactive oxygen species (ROS), malondialdehyde (MDA) of lipid peroxidation, and intracellular Fe2+ level in BC cells. Furthermore, treatment with curcumin significantly suppresses tumorigenesis in BC via upregulating SLC1A5 expression, which is an essential transporter for glutamine uptake.

As a whole, the authors performed thorough experimental plan, and their finding are quite interesting. However, the authors need further improvement of this article to grown-up to publishing-quality. The followings are the issues that the authors should be addressed.

**Question #1:** Because there are some other published articles in which Li R, et al, revealed the anti-tumorigenic effect of curcumin by altering other ferroptosis associated gene (HO-1) in breast cancer area (Oxid Med Cell Longev. 2020 Nov 18; 2020:3469840), the authors should add this article as reference and should add some discussion with regard to this article.

**Response #1:** As suggested, we cited this reference as Ref.39 and added some discussion in line 304-308, page 12 in this revision.

**Question #2:** As for Figure2A,2B, and 3F, please add the title of X-axis. Lipid ROS level?

**Response #2:** As suggested, we added the title of X-axis as “Lipid ROS content” in Figure 2A, 2B and 3F.

**Question #3:** How about changing the position of Figure 5F to 5A in order to explain the less-toxic effect of curcumin to mice?

**Response #3:** As suggested, we exchanged the position of Figure 5F and 5A.

**Reviewer #3**

In this article, the authors show that curcumin exhibits anti-tumorigenesis activity in breast cancer cell lines by promoting SLC1A5-mediated ferroptosis (lipid ROS, lipid peroxidation end-product MDA accumulation, and intracellular Fe2+ levels). Objectives are clear, experiments are well designed, and methodology is adequate to obtain the results. The article is well written.

**Major issues**

**Question #1:** Which diluent is used to dissolve curcumin? It must be checked that the vehicle does not reduce cells viability.

**Response #1:** As suggested, we provided the dilution method of curcumin and test the cell viability of DMSO solvent. As shown in supplementary Fig. 1, the corresponding volume of DMSO had no cytotoxicity on MDA-MB-453 and MCF-7 cells (supplementary Fig. 1). (Line 86-88, page 4 and line 189-191, page 8 in this revision.)

**Question #2:** Authors explain the role of ASCT2, SLC1A5 and SLC38A1 in glutamine uptake, but they only measured SLC1A5. Then they argue about the role of SLC1A5 in ferroptosis induced by curcumin but not about other receptors. Other receptors should be measured to confirm SLC1A5 implication.

Response #2: ASCT2 is encoded by SLC1A5 that was evaluated in this original manuscript. As suggested, we further confirmed the mRNA expression of SLC38A1 in MDA-MB-453 and MCF-7 cells after treated with or without curcumin. As shown in supplementary Fig. 3, curcumin significantly enhanced the expression SLC1A5, but had no obvious effect on the expression of SLC38A1. We added this information in line 221-222, page 9 in this revision.

**Question #3:** Immunohistochemistry staining should be quantified; a representative image is not enough to conclude Ki-67 and SLC1A5 increase.

**Response #3:** As suggested, we provided the quantified results of Immunohistochemistry staining as Figure 5F in this revision.

**Question #4:** To confirm the role of ferroptosis in vivo, authors should perform the same experiment but treating mice with ferrostatin and other cell death inhibitors, such as ZVAD and NS. In addition, authors should measure other ferroptosis markers than just MDA in the tumor to conclude the role of this pathway.

**Response #4:** As suggested, to confirm that curcumin inhibited tumor growth in *vivo* by ferroptosis, we treated mice with curcumin, curcumin + DFO and curcumin + NS. As shown in Figure 5B-D in this revision, the administration of curcumin + DFO obviously reversed the inhibitory effect of curcumin on tumor growth, whereas the addition of NS, did not impact the efficiency of curcumin, which was consistent with the results of in *vitro* experiments that curcumin showed an antitumor effect on BC cells by inducing cell ferroptosis. (Line 248-255, page 10 in this revision.)

In addition, we supplemented the results of other ferroptosis markers such as GSH and iron in tumor tissues of mice and the results were shown in Figure 5G-I. (Line 259-262, page 10 in this revision.)

**Question #5:** In the discussion sections, authors report that curcumin promotes ferroptosis. However, there are contradictory data in the bibliography about this issue, see reference 38 (Guerrero-Hue et al.). Authors should discuss about the possible dual role of curcumin on ferroptosis.

**Response #5:** Thanks for your comments, we have supplemented the possible dual role of curcumin on ferroptosis in the Discussion section. (Line 304-308, page 12 in this revision.)

**Question #6:** Authors should add in the figure legend the time in which each experiment was performed.

**Response #6:** As suggested, we added the treatment time in which each experiment in the figure legend. (Line 474, 476, 482 and 508, page 17-18 in this revision.)

**References in this letter:**

1.	Mohammed F, Rashid-Doubell F, Taha S, Cassidy S, Fredericks S. Effects of curcumin complexes on MDAMB231 breast cancer cell proliferation. Int J Oncol. 2020;57(2):445-55. Epub 2020/07/07. doi: 10.3892/ijo.2020.5065. PubMed PMID: 32626932; PubMed Central PMCID: PMCPMC7307592.

2.	Hu CX, Li MJ, Guo TT, Wang SX, Huang WP, Yang K, et al. Anti-metastasis activity of curcumin against breast cancer via the inhibition of stem cell-like properties and EMT. Phytomedicine. 2019;58. doi: ARTN 152740

10.1016/j.phymed.2018.11.001. PubMed PMID: WOS:000473047100001.

3.	Xiong K, Zhang Y, Wen Q, Luo J, Lu Y, Wu Z, et al. Co-delivery of paclitaxel and curcumin by biodegradable polymeric nanoparticles for breast cancer chemotherapy. Int J Pharm. 2020;589:119875. Epub 2020/09/13. doi: 10.1016/j.ijpharm.2020.119875. PubMed PMID: 32919003.

4.	Kalluru H, Kondaveeti SS, Telapolu S, Kalachaveedu M. Turmeric supplementation improves the quality of life and hematological parameters in breast cancer patients on paclitaxel chemotherapy: A case series. Complement Ther Clin Pract. 2020;41:101247. Epub 2020/10/26. doi: 10.1016/j.ctcp.2020.101247. PubMed PMID: 33099272.

5.	Wen C, Fu L, Huang J, Dai Y, Wang B, Xu G, et al. Curcumin reverses doxorubicin resistance via inhibition the efflux function of ABCB4 in doxorubicinresistant breast cancer cells. Mol Med Rep. 2019;19(6):5162-8. Epub 2019/05/07. doi: 10.3892/mmr.2019.10180. PubMed PMID: 31059026; PubMed Central PMCID: PMCPMC6522915.
