# Supplementary material for: Curcumin suppresses tumorigenesis by ferroptosis in breast cancer
Source: PLoS One. 2022 Jan 18;17(1):e0261370. doi: 10.1371/journal.pone.0261370 (PMC8765616; doi:10.1371/journal.pone.0261370)
Supplement: Attachment — Submitted filename: Response to Reviewers.docx [file pone.0261370.s006.docx]

**Response to reviewer**

**Reviewer #1:**

**Question #4/Response #4(1):** The authors did not discuss the chelating ability of curcumin and ignored the related references (Ak and Guicin, 2008; Hirata et al., 2020, Shome et al., 2021). The chelating ability of curcumin is opposite action the authors reported in this study and therefore the authors should discuss the discrepancy.

The authors examined the effect of deferoxamine (DFO) on the curcumin-induced increase in intracellular Fe2+ (Figure S2). DFO only affected curcumin-induced increase in intracellular ferrous ions. The results are strange. Why DFO did not decrease the control level of ferrous ions?

**Response:** We felt great thanks for your professional review work on our article. As suggested, we added the related references (Ak and Guicin, 2008; Hirata et al., 2020, Shome et al., 2021) as Ref. 41-43 and discussed the discrepancy between these studies and our work (line 308-318, page 12 in this revision).

In addition, as shown in supplementary **Fig. 2**, our data showed that DFO did not only affect curcumin-induced increase in intracellular ferrous ions, but also decrease the control level of ferrous ions (the second column).

**Question #4/Response #4(2):** The authors correct the unit of ferrous ion μg/μg protein to nmol/μg protein. According to the abcam’s protocol (ab83366, abcam) the authors used, rat liver lysate contains approximately 0.1 nmoles Fe2+/mg tissue, which is approximately 10,000 times less compared the amount of Fe2+ the authors reported (10 µmoles/µg protein).

**Response:** In this study, the unit of ferrous was (nmol/μg protein) × 10^3^ in Fig. 2 and 3, which is equivalent to nmol/mg protein.

**Question #5/Response #5:** Erastin at 10 nM cannot cause ferroptotic cell death in cultured cells. Most cancer cells are required 5-10 µM erastin to cause ferroptosis.

**Response:** We are very sorry for our carelessness in writing. After careful checking, we verified that the dose of erastin used in this experiment was 10 µM. Special thank you very much for your careful review and we express our sincere apologies for this error. We corrected this mistake in this revision (line 90, page 4 and line 501, page 18 in this revision).
