# Supplementary material for: Curcumin suppresses tumorigenesis by ferroptosis in breast cancer
Source: PLoS One. 2022 Jan 18;17(1):e0261370. doi: 10.1371/journal.pone.0261370 (PMC8765616; doi:10.1371/journal.pone.0261370)
Supplement: Attachment — Submitted filename: Response to Reviewers.docx [file pone.0261370.s007.docx]

**PONE-D-21-20659R2**

**Response to reviewer**

**Reviewer #1:**
**Question #1:** The authors did not respond my concern as follows:
DFO only affected curcumin-induced increase in intracellular ferrous ions (Figure S2A, the 3rd column vs 4th column) but did not affect the control level of ferrous ions (Figure S2A, the first column vs second column). The results are strange.

**Response #1:** We retest the intracellular ferrous ions in MDA-MB-453 and MCF-7 cells with or without the presence of DFO. As shown in Figure 1 in this response, no obvious difference was observed between the intracellular ion level in of MDA-MB-453 and MCF-7 cells with or without the presence of 50 μM deferoxamine, which was also found in recent published researches [1-3]. Although deferoxamine mesylate was an iron chelator that bound free iron in a stable complex, we speculated that it might not affect the relatively low normal ferrous ions level in some cells but effectively reverse the iron accumulation induced by ferroptosis inducer.

**Figure 1.** Intracellular Fe^2+^ level of MDA-MB-453 and MCF-7 cells with or without the presence of 50 μM deferoxamine (DFO).

**Question #2:** The English writing standard needs to be improved further.

**Response #2:** As suggested, we improved the quality of our language by an English language editing service of Spandidos Publications. Based on the editing report, we revised our manuscript and also uploaded the certificate with this revision.

**References in this response:**

1.	Tu H, Zhou YJ, Tang LJ, Xiong XM, Zhang XJ, Ali Sheikh MS, et al. Combination of ponatinib with deferoxamine synergistically mitigates ischemic heart injury via simultaneous prevention of necroptosis and ferroptosis. Eur J Pharmacol. 2021;898:173999. Epub 2021/03/07. doi: 10.1016/j.ejphar.2021.173999. PubMed PMID: 33675785.

2.	Yang J, Zhou Y, Xie S, Wang J, Li Z, Chen L, et al. Metformin induces Ferroptosis by inhibiting UFMylation of SLC7A11 in breast cancer. J Exp Clin Cancer Res. 2021;40(1):206. Epub 2021/06/25. doi: 10.1186/s13046-021-02012-7. PubMed PMID: 34162423; PubMed Central PMCID: PMCPMC8223374.

3.	Song Z, Xiang X, Li J, Deng J, Fang Z, Zhang L, et al. Ruscogenin induces ferroptosis in pancreatic cancer cells. Oncol Rep. 2020;43(2):516-24. Epub 2020/01/03. doi: 10.3892/or.2019.7425. PubMed PMID: 31894321; PubMed Central PMCID: PMCPMC6967081.
